# PML/TRIM19-Dependent Inhibition of Retroviral Reverse-Transcription by Daxx

**DOI:** 10.1371/journal.ppat.1005280

**Published:** 2015-11-13

**Authors:** Jacques Dutrieux, Ghizlane Maarifi, Débora M. Portilho, Nathalie J. Arhel, Mounira K. Chelbi-Alix, Sébastien Nisole

**Affiliations:** 1 Paris Descartes University, Paris, France; 2 University Institute of Hematology, Saint-Louis Hospital, Paris, France; Vanderbilt University School of Medicine, UNITED STATES

## Abstract

PML (Promyelocytic Leukemia protein), also known as TRIM19, belongs to the family of tripartite motif (TRIM) proteins. PML is mainly expressed in the nucleus, where it forms dynamic structures known as PML nuclear bodies that recruit many other proteins, such as Sp100 and Daxx. While the role of PML/TRIM19 in antiviral defense is well documented, its effect on HIV-1 infection remains unclear. Here we show that infection by HIV-1 and other retroviruses triggers the formation of PML cytoplasmic bodies, as early as 30 minutes post-infection. Quantification of the number and size of PML cytoplasmic bodies revealed that they last approximately 8 h, with a peak at 2 h post-infection. PML re-localization is blocked by reverse-transcription inhibitors and is not observed following infection with unrelated viruses, suggesting it is specifically triggered by retroviral reverse-transcription. Furthermore, we show that PML interferes with an early step of retroviral infection since PML knockdown dramatically increases reverse-transcription efficiency. We demonstrate that PML does not inhibit directly retroviral infection but acts through the stabilization of one of its well-characterized partners, Daxx. In the presence of PML, cytoplasmic Daxx is found in the vicinity of incoming HIV-1 capsids and inhibits reverse-transcription. Interestingly, Daxx not only interferes with exogenous retroviral infections but can also inhibit retrotransposition of endogenous retroviruses, thus identifying Daxx as a broad cellular inhibitor of reverse-transcription. Altogether, these findings unravel a novel antiviral function for PML and PML nuclear body-associated protein Daxx.

## Author Summary

Among the several protection mechanisms raised by mammalian organisms against viral infections, an early line of defense consists of cellular proteins known as restriction factors that interfere with viral replication. PML, also known as TRIM19, is one of these proteins and has been shown to inhibit diverse viruses. PML is mainly expressed in the nucleus, where it forms dynamic structures known as PML nuclear bodies that recruit many other proteins, such as Sp100 and Daxx. While the role of PML/TRIM19 in antiviral defense is well documented, its effect on HIV-1 infection remains unclear. Here we show that PML is rapidly re-localized in the cytoplasm of cells infected by HIV-1 and other retroviruses and interferes with reverse-transcription of incoming retroviral RNA. We were able to demonstrate that PML does not inhibit retroviral infection directly but through the stabilization of one of its well-characterized partners, Daxx. In the presence of PML, Daxx inhibits an early step of reverse-transcription thereby interfering with retroviral infections. Our findings unravel a novel antiviral function for PML and its partner Daxx.

## Introduction

Intrinsic immunity constitutes the first line of defense against viral infection, as it relies on cellular proteins, known as restriction factors, to directly counteract viral replication. An increasing number of restriction factors targeting various steps of HIV-1 replication have been identified during the last decade. TRIM5α is one of these factors. It was originally identified as the protein responsible for HIV-1 restriction in rhesus macaque cells [1]. TRIM5α was later found to constitute a species-specific barrier to various retroviruses in other primates, including humans [2–4]. TRIM5α belongs to a vast family of proteins known as tripartite motif (TRIM) proteins, as they share a common RBCC motif composed, from N- to C-terminus, by a RING domain, one or two B-Boxes and a coiled-coil domain [5,6]. Interestingly, among the 70 members identified in human cells, TRIM5α is not the only TRIM protein with the capacity to interfere with HIV-1 replication [7–10]. TRIM22 for example, was shown to interfere both with HIV-1 transcription [7] and assembly [8]. Unlike TRIM5α whose antiviral activity only targets retroviruses, TRIM22 also confers resistance to hepatitis B virus (HBV) [11], Encephalomyocarditis virus (EMCV) [12] and Influenza A virus [13]. Another TRIM protein that displays a broad intrinsic antiviral activity is TRIM19, better known as PML, for Promyelocytic Leukemia protein. PML was shown to interfere with the replication of many unrelated viruses, including human foamy virus (HFV) [14], poliovirus [15], influenza virus [16], rabies virus [17], EMCV [18], adeno-associated virus (AAV) [19] and vesicular stomatitis virus (VSV) [20]. PML was also proposed to interfere with an early step of HIV infection, since arsenic trioxide (As_2_O_3_), a chemical compound known to promote PML degradation, was found to increase HIV transduction efficiency [21]. Interestingly, Turelli *et al* also showed that HIV-1 infection induces a rapid translocation of PML from the nucleus to the cytoplasm, where it colocalizes with INI1 and HIV pre-integration complexes. However, the finding that As_2_O_3_-induced enhancement of HIV-1 replication was independent of its effect on PML cast some doubts on PML's anti-HIV activity [22]. PML is expressed in human cells as 7 different isoforms, named PMLI to PMLVIIb, generated by alternative splicing from a single *PML* gene [23]. All isoforms share the same N-terminal portion, composed of the RBCC motif, but differ from each other by their C-terminal region. Since PMLVIIb is devoid of a nuclear localization signal, it is found exclusively in the cytoplasm. In contrast, PMLI to PMLVI are localized in the nucleus, both diffuse in the nucleoplasm and within punctate domains known as PML nuclear bodies (PML NBs). PML NBs are dynamic structures and PML is essential for their formation [24]. A plethora of proteins have been shown to be recruited by PML within PML NBs, either permanently, such as death-domain-associated protein (Daxx), Sp100 and SUMO, or transiently, such as p53 or CAMP response element–binding protein (CBP). PML NBs are implicated in many cellular processes including cell cycle progression, DNA damage response, transcriptional regulation and apoptosis [25,26]. In addition, several PML NB components have been implicated in antiviral defense and are interferon-stimulated gene (ISG) products, including PML itself, Daxx and Sp100 [27]. As a direct consequence, many viruses have developed strategies to counteract PML NB repression by disrupting PML NBs and/or by inducing PML degradation [27,28].

In this study, we re-evaluated the involvement of PML during HIV-1 infection and provide new insight into its involvement in retroviral restriction. We confirm that HIV-1 infection triggers a rapid re-localization of PML, leading to the appearance of PML bodies in the cytoplasm. Importantly, PML re-localization is correlated with a significant decrease in HIV-1 transduction, an observation suggesting that PML could interfere with an early step of HIV-1 replication. Furthermore, we show that other retroviruses also trigger PML cytoplasmic localization and show increased transduction efficiency in the absence of PML. In contrast, no PML could be detected in the cytoplasm of cells infected with viruses belonging to other viral families, suggesting that the appearance of cytoplasmic PML correlates with restriction. However, we show that even while PML is required, it is not the direct mediator of retroviral restriction. Indeed, we show that one of PML's well-known interacting partners, Daxx, is the restriction factor that is affected by PML depletion. Daxx interferes with reverse-transcription (RT) and is thus able to inhibit retroviral infections but also retro-transposition events. Finally, we deciphered the exact role of PML in Daxx-mediated retroviral restriction by showing that PML stabilizes Daxx, which is rapidly degraded in PML knockdown cells. Altogether, our study confirms the involvement of PML in retroviral infection and reveals Daxx as a novel retroviral restriction factor targeting RT.

## Results

### Human PML interferes with an early step of HIV cycle

**Fig 1:**
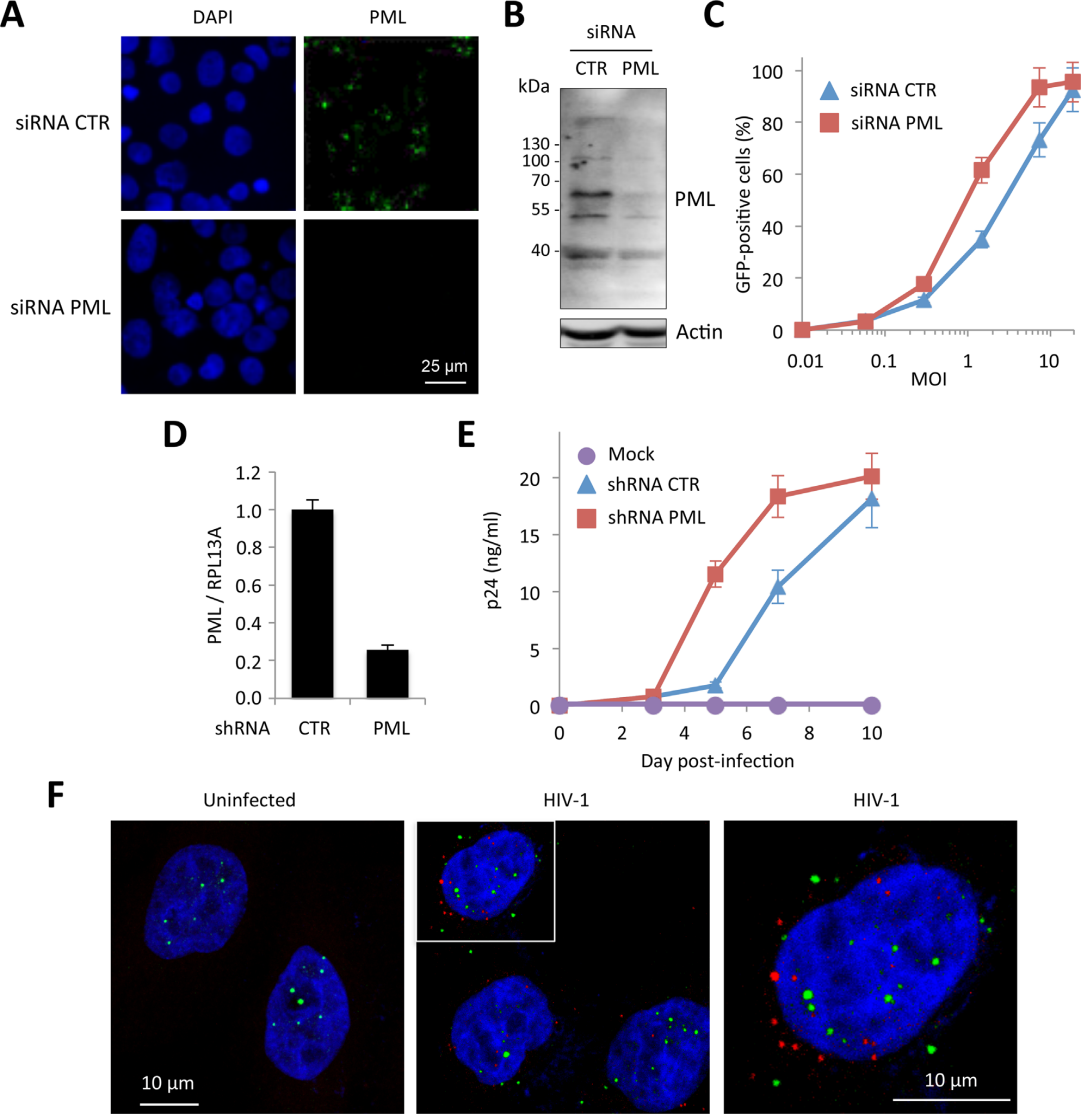
PML expression in human cells interferes with HIV infection. (A and B) HeLa cells were transfected with siRNA directed against all PML isoforms or a non-targeting siRNA (CTR). Knockdown efficiency was estimated by immunofluorescence (A) and Western-blot (B). (C) Cells were then infected with VSV-G pseudotyped HIV-1 vector expressing GFP at increasing MOI during 48h and the percentage of GFP expressing cells was determined by flow cytometry. The graph shows means +/- SD of 3 independent experiments performed in duplicates (D and E) PBMCs of healthy adult individuals were activated by anti-CD3/anti-CD28 for 48 h and were subsequently transduced with a lentiviral vector expressing either a non-targeting control (CTR) or a PML-specific shRNA. (D) Relative expression of PML was estimated by RT-qPCR analyis performed on PBMCs two days post-transduction, and expressed as a ratio to RPL13A mRNA expression. (E) Two days post transduction with the shRNA-expressing vector, activated PBMCs were infected with HIV-1 NL4.3 at a dose corresponding to 0.1 ng of p24 and virus production was measured at given time points by measuring the amount of HIV-1 p24 by ELISA on the culture supernatants. Results show the mean of triplicates +/- SD and are representative of two independent experiments. (F) HeLa wt cells were transduced at MOI 10 with HIV-1 derived vector during 4 h and PML (in green) and HIV-1 p24 (in red) were detected by immunofluorescence.

First, we tested the effect of PML knockdown on HIV infection. HeLa cells were transfected with siRNA directed against all PML isoforms and challenged with a GFP encoding HIV-1 vector pseudotyped with VSV-G envelop. The efficacy of PML knockdown was estimated both by immunostaining (Fig 1A) and immunoblot (Fig 1B). PML depletion led to a modest but reproducible increase in the proportion of transduced cells, from around 30 to 60% at MOI = 0.5 (Fig 1C). This first observation, although tentative, is in favor of a negative effect of endogenous PML on HIV early steps of replication. Next, we tested the effect of PML knockdown on HIV-1 propagation in primary cells. For this, PBMCs from healthy donors were first transduced with a retroviral vector expressing a shRNA targeting PML or a non-targeting shRNA (CTR), and infected 48 h later with 0.1 ng p24 of HIV-1 NL4.3. RT-qPCR analysis performed two days post-transduction revealed that PML mRNA expression was reduced by approximately 5-fold (Fig 1D). As shown in Fig 1E, HIV-1 spreading infection was significantly enhanced in PML-depleted cells, further confirming that PML expression interferes with HIV-1 infection.

Next, we investigated the fate of PML proteins in HIV-transduced cells by immunofluorescence (Fig 1F). As early as 4 h post-transduction, we observed the appearance of PML dots in the cytoplasm, suggesting that HIV infection induced a translocation of PML from the nucleus to the cytoplasm. However, we observed no or very little co-localization between PML cytoplasmic bodies (CBs) and incoming HIV-1 cores labeled using anti-p24 antibodies (Fig 1F).

### Anti-HIV activity of PML in murine cells

**Fig 2:**
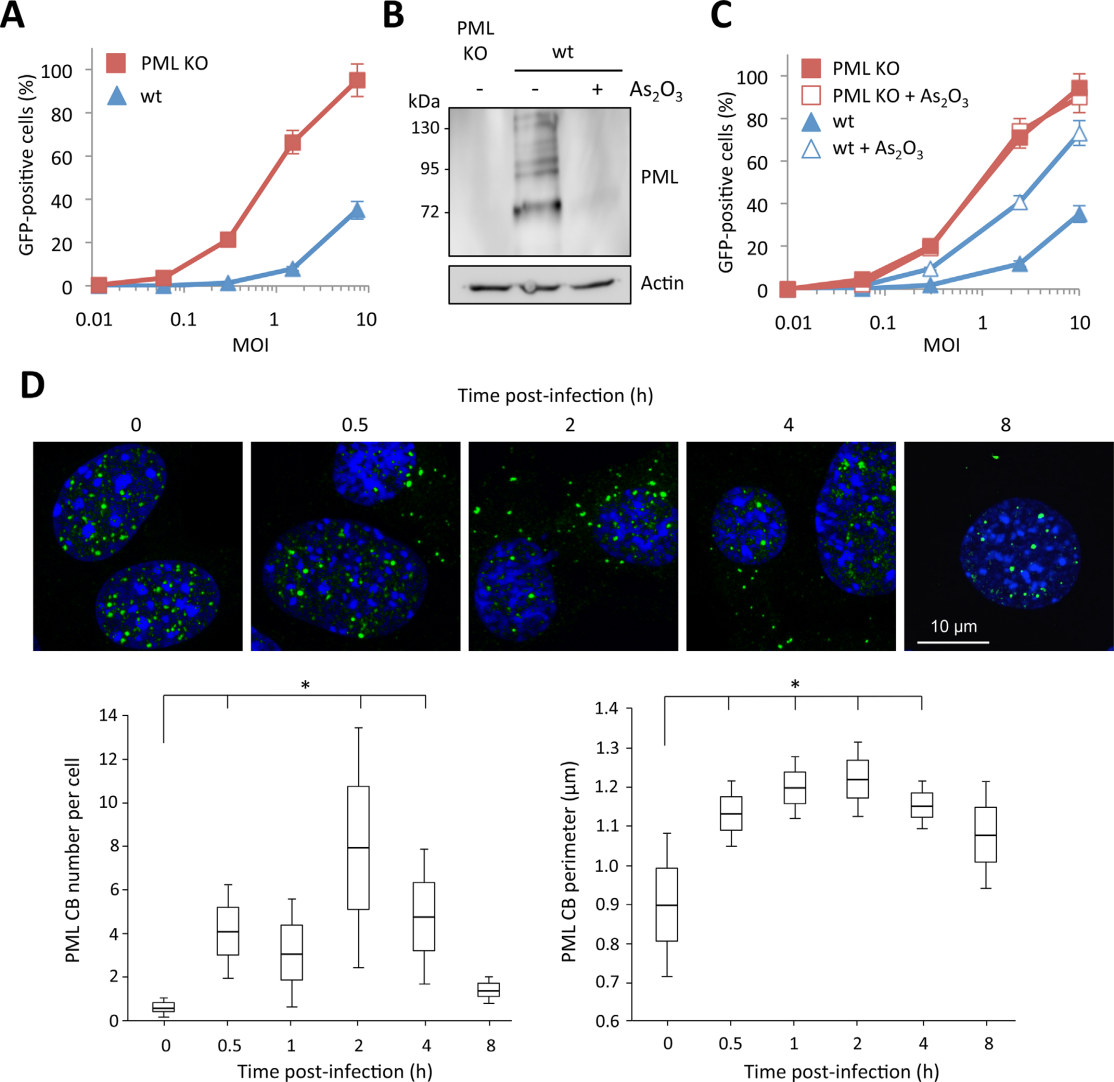
Role of murine PML in HIV-1 transduction. (A) Wt or PML KO MEFs were transduced at different MOI with HIV-1 derived lentivector expressing GFP during 48h and the percentage of GFP expressing cells was determined by flow cytometry. The graph shows means of duplicate values +/- SD, representative of three independent experiments. (B and C) Wt or PML KO MEFs were treated with 2.5 μM of As_2_O_3_ during 24h, PML degradation was evaluated by western-blot (B) and cells were infected at different MOI with HIV-1 vector during 48h and the percentage of GFP expressing cells was determined by flow cytometry (C). The graph shows means of duplicate values +/- SD, representative of three independent experiments. (D) Wt MEFs were infected for different times at MOI 10 with HIV-1 derived vector and PML localization was determined by immunofluorescence. For each time-point, the number and perimeter of PML CBs was quantified in 50 cells from 5 independent fields. Student t-test was performed to determine P values. (*P<0.05).

In order to confirm the involvement of PML in HIV-1 restriction, we compared the capacity of the HIV-1 vector to transduce mouse embryonic fibroblasts (MEFs) derived from wild-type (wt) and PML KO mice [29]. Strikingly, the difference in the proportion of GFP-positive cells was even more pronounced than in human cells, since at a MOI of 10, only 30% of wt MEFs were transduced, while over 90% of PML KO MEFs were GFP-positive (Fig 2A). Since the anti-HIV activity of PML was more potent in murine than in human cells, we carried out further experiments using this model.

Arsenic trioxide (As_2_O_3_) increases PML SUMOylation and promotes its proteasomal degradation through its recognition by RNF4, a poly-SUMO-dependent ubiquitin E3 ligase [30,31]. We treated wt MEFs with this chemical compound and transduced them with the HIV-GFP vector. As expected, As_2_O_3_ treatment led to PML degradation (Fig 2B) and increased the capacity of wt MEFs to be transduced (Fig 2C), further suggesting a potent anti-HIV activity of PML. In contrast, As_2_O_3_ had no effect on HIV transduction of PML KO MEFs, thus confirming that the effect of this agent is mediated through PML degradation.

Next, we sought to determine whether HIV-1 transduction triggers the appearance of PML in the cytoplasm of murine cells, as we observed in human cells. For this, we performed a time-course of HIV-1 transduction, from 0.5 to 8 h and followed PML localization by confocal microscopy (Fig 2D). As expected, HIV-1 also induced the appearance of PML dots in the cytoplasm of wt MEFs. Surprisingly, these cytoplasmic PML bodies can be detected as early as 0.5 h post-transduction, whereas they can no longer be detected after 8 h. Quantification of the number and size of PML CBs (Fig 2D) revealed that the cytoplasmic localization of PML following HIV-1 infection lasts less than 8 h. The peak in term of number and size of PML CBs occurred at 2 h post-transduction, with an average of 8 PML CBs per cell (Fig 2D).

### PML, reverse-transcription and SUMO

**Fig 3:**
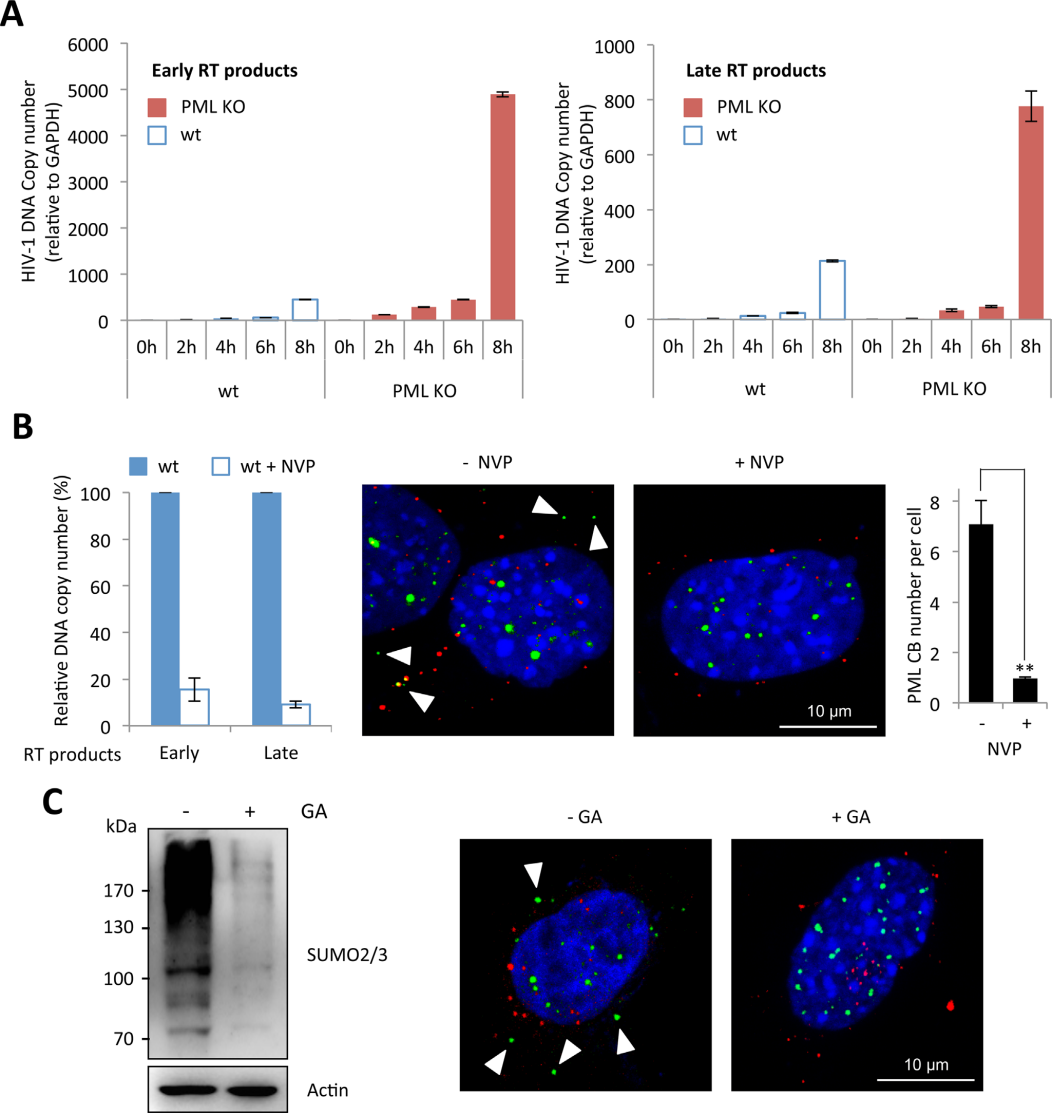
Reverse-transcription is the step affected by PML expression and the required event triggering PML CB formation. (A) Wt or PML KO MEFs were transduced for different times at MOI 10 with VSV-G pseudotyped HIV-1 derived vector, DNA was extracted and early and late reverse transcript were quantified by qPCR. (B) Wt or PML KO MEFs were treated for 30 min with nevirapine (NVP) before a 2 h transduction at MOI 10 with HIV-1 vector. DNA was extracted and early and late RT products were quantified by qPCR (left panel). PML (in green) and p24 (in red) localization was determined by immunofluorescence (middle panel). For each condition, the number of PML CBs was quantified in 50 cells from 3 independent fields (right panel). Student t-test was performed to determine P values (**P<0.01). (C) The amount of SUMOylated proteins in wt MEFs treated with GA for 24 h was evaluated by western-blot (left panel). In parallel, wt MEFs pretreated with GA for 4 h were transduced for 2 h at MOI 10 with HIV-1 vector and PML (in green) and HIV-1 p24 (in red) localization was determined by immunofluorescence (right panel).

Next, we investigated the precise step of HIV replicative cycle that is enhanced following PML knockdown in murine cells. To do this, we performed a kinetic analysis of HIV transduction in wt and PML KO MEFs and determined the amount of early and late RT products by quantitative PCR. As shown in Fig 3A, the amount of RT products was much higher in PML KO compared to wt MEFs, suggesting that PML expression prevents the accumulation of RT products. We therefore asked whether RT triggers PML re-localization to the cytoplasm. We treated wt MEFs with nevirapine (NVP), a non-nucleoside RT inhibitor and transduced them with HIV-1. As expected, NVP treatment led to a dramatic reduction of both early and late RT products, quantified by quantitative PCR (Fig 3B, left panel). Interestingly, whereas PML bodies can be detected in the cytoplasm of untreated cells 2 h post-transduction, no PML CBs could be detected in NVP-treated cells (Fig 3A, right panel), indicating that RT is a prerequisite for the formation of PML CBs.

Since the formation of PML NBs is known to depend on PML SUMOylation [24,32], we investigated whether this modification is also required for the formation of PML CBs upon HIV-1 infection. For this purpose, wt MEFs were pre-treated or not with Ginkgolic acid (GA), a compound that specifically inhibits the formation of the E1-SUMO intermediates, thus preventing SUMOylation [33], and transduced with HIV-1 vector. We verified by western-blot using SUMO2/3 antibodies that the amount of SUMOylated proteins was dramatically reduced after 24h of GA treatment (Fig 3C, left panel). Under these conditions, we did not observe any PML body in the cytoplasm upon HIV-1 transduction. Although this does not formally prove that PML SUMOylation is required for its change of localization and/or for the formation of PML CBs, it demonstrates that SUMOylation is involved.

### Effect of PML knockdown on other viruses

**Fig 4:**
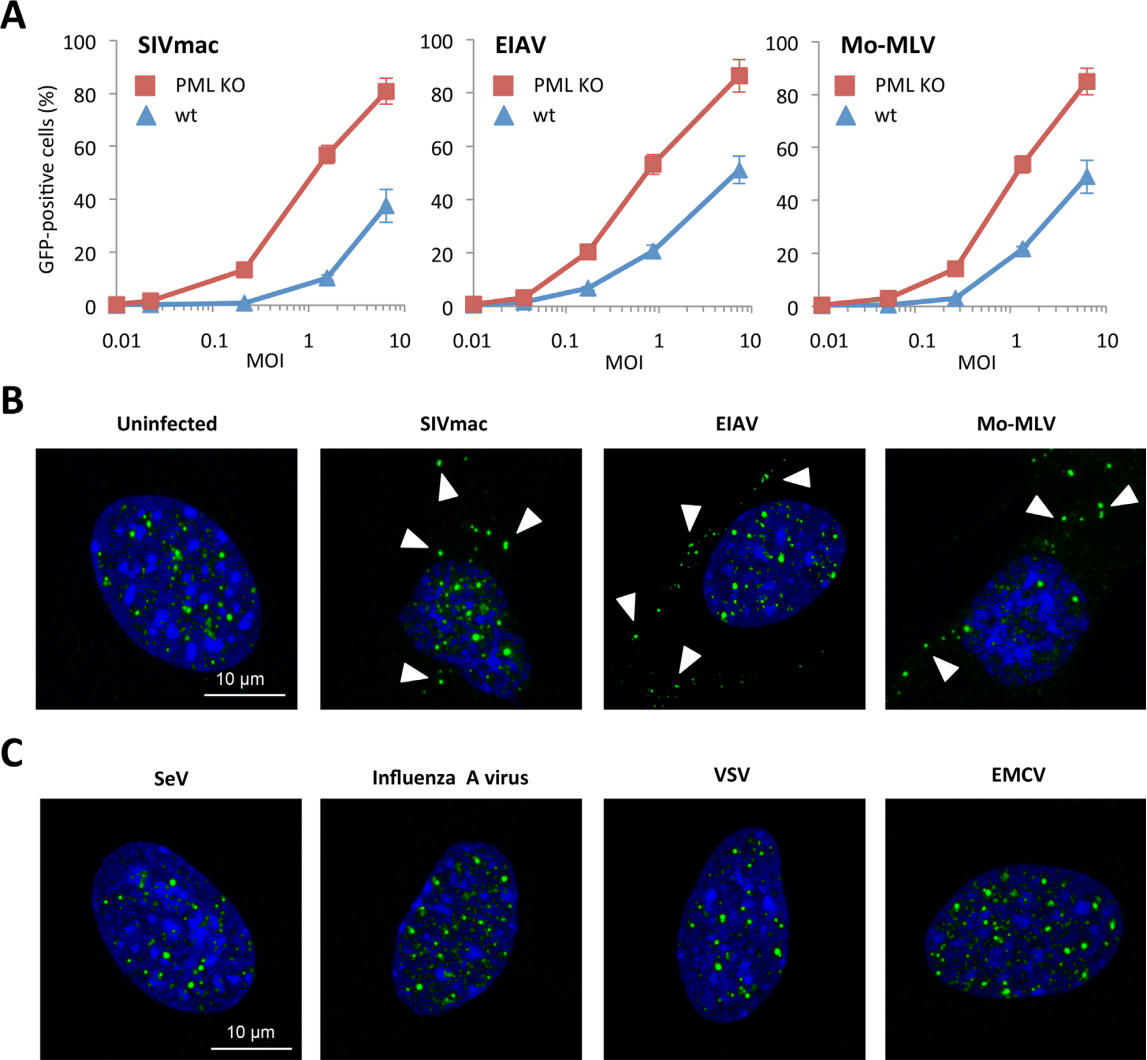
Effect of PML expression on retroviral infection. (A) Wt or PML KO MEFs were transduced at different MOI with VSV-G pseudotyped EIAV, Moloney-MLV or SIVmac derived vectors expressing GFP during 48h and the percentage of GFP expressing cells was determined by flow cytometry. The graph shows means of triplicate values +/- SD, representative of three independent experiments. (B) Wt MEFs were transduced for 2 h at MOI 5 with EIAV, Moloney-MLV or SIVmac derived vectors and PML localization was determined by immunofluorescence. Arrows point to representative cytoplasmic PML dots. (C) Wt MEFs were infected for 2 h at MOI 10 with SeV, Influenza A virus, VSV or EMCV and PML localization was determined by immunofluorescence.

In order to determine whether PML can only restrict HIV-1 or also other retroviruses, we transduced wt or PML KO MEFs with increasing doses of VSV-G pseudotyped SIVmac, EIAV or MLV Moloney (Mo-MLV). As observed in the case of HIV-1, the transduction efficiency of PML KO MEFs was higher than wt cells for SIVmac, EIAV and Mo-MLV (Fig 4A). This result suggests that PML has a broad anti-retroviral activity that is not limited to HIV-1.

Next, we investigated PML localization in wt MEFs transduced with SIVmac, EIAV and Mo-MLV. All retroviruses were able to trigger the formation of PML CBs (Fig 3B). We also performed infections with viruses unrelated to the *retroviridae* family, in order to determine whether the formation of PML CBs was specific to retroviral infections. For this, wt MEFs were infected for 4 h with Sendai virus (SeV), Influenza A virus, VSV or EMCV. As observed in Fig 4D, no cytoplasmic PML staining could be detected, indicating that the formation of PML CBs is a specific event occurring only upon retroviral infections (Fig 4D).

### PML is required but not sufficient to promote HIV-1 restriction

**Fig 5:**
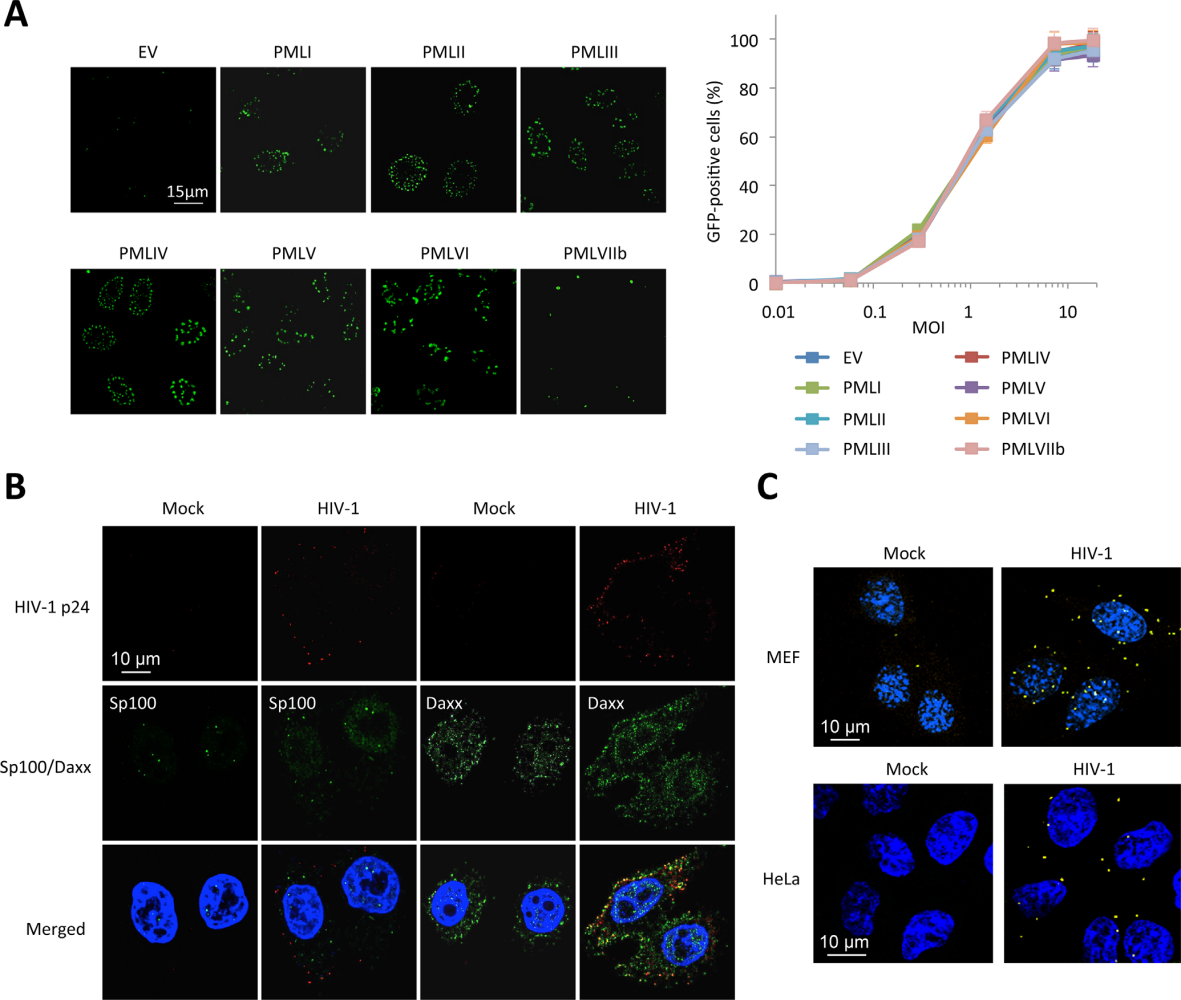
PML is not the main mediator of HIV-1 restriction. (A) PML KO MEFs were transduced with HIV-1 derived lentiviral vectors expressing each human PML isoform (PMLI to PMLVIIb) or with an empty vector (EV). PML isoform expression was evaluated by immunofluorescence. Two days post-transduction, PML expressing cells were transduced with GFP-expressing HIV-1 vector and the percentage of GFP-expressing cells was evaluated by flow cytometry. Graph shows means of triplicate values +/- SD, representative of two independent experiments. (B) Wt HeLa cells were transduced for 2 h with HIV-1 vector and HIV-1 p24 (in red), Sp100 and Daxx (in green) localization was analyzed by immunofluorescence. (C) Wt MEFs or HeLa cells were left untransduced or transduced with HIV-1 for 2 h and probed with anti-Daxx and anti-HIV-1 p24 antibodies. Representative images of a Duolink experiment are shown.

Since all our results are in favor of an inhibition of RT by PML, we decided to determine which PML isoform was responsible. We transduced PML KO MEFs with retroviral vectors expressing each one of the seven human isoforms of PML (PMLI to VIIb). Immunofluorescence analyses confirmed that PML isoforms I to VI are found within PML NBs whereas PMLVIIb forms dots in the cytoplasm (Fig 5A, left panel), as anticipated [18]. These cells were transduced with HIV-1 and the percentage of transduced cells was estimated by flow cytometry 48 h post-transduction. Unexpectedly, none of the overexpressed human PML isoforms conferred resistance to HIV-1 in MEFs. (Fig 5A, right panel). This result led us to suspect that, even if PML is involved in HIV-1 restriction, it might not be the direct mediator of restriction.

We therefore looked at the localization of proteins that are constitutively associated to PML within PML NBs, Sp100 and Daxx (Fig 5B). Whereas Sp100 is found exclusively in the nucleus of uninfected cells, some Sp100 dots could be observed in the cytoplasm 2 h post-HIV-1 transduction, but similarly to PML did not colocalize with HIV-1 p24. In contrast, whereas Daxx is mainly nuclear, it is also expressed in the cytoplasm in uninfected cells. Following transduction with HIV-1 for 2 h, many Daxx cytoplasmic dots were found to colocalize with HIV-1 p24 (Fig 5B). To confirm the close vicinity of Daxx with incoming HIV-1 cores, we performed Duolink *in situ* proximity ligation assay (PLA). Cells were probed with anti-Daxx and anti-HIV-1 p24 primary antibodies, followed by species-specific PLA probes. Our results confirm a close proximity of Daxx with incoming HIV-1 capsids (Fig 5C), thus suggesting that Daxx might be implicated in PML-dependent HIV-1 restriction.

### Daxx is the cellular protein responsible for retroviral restriction

**Fig 6:**
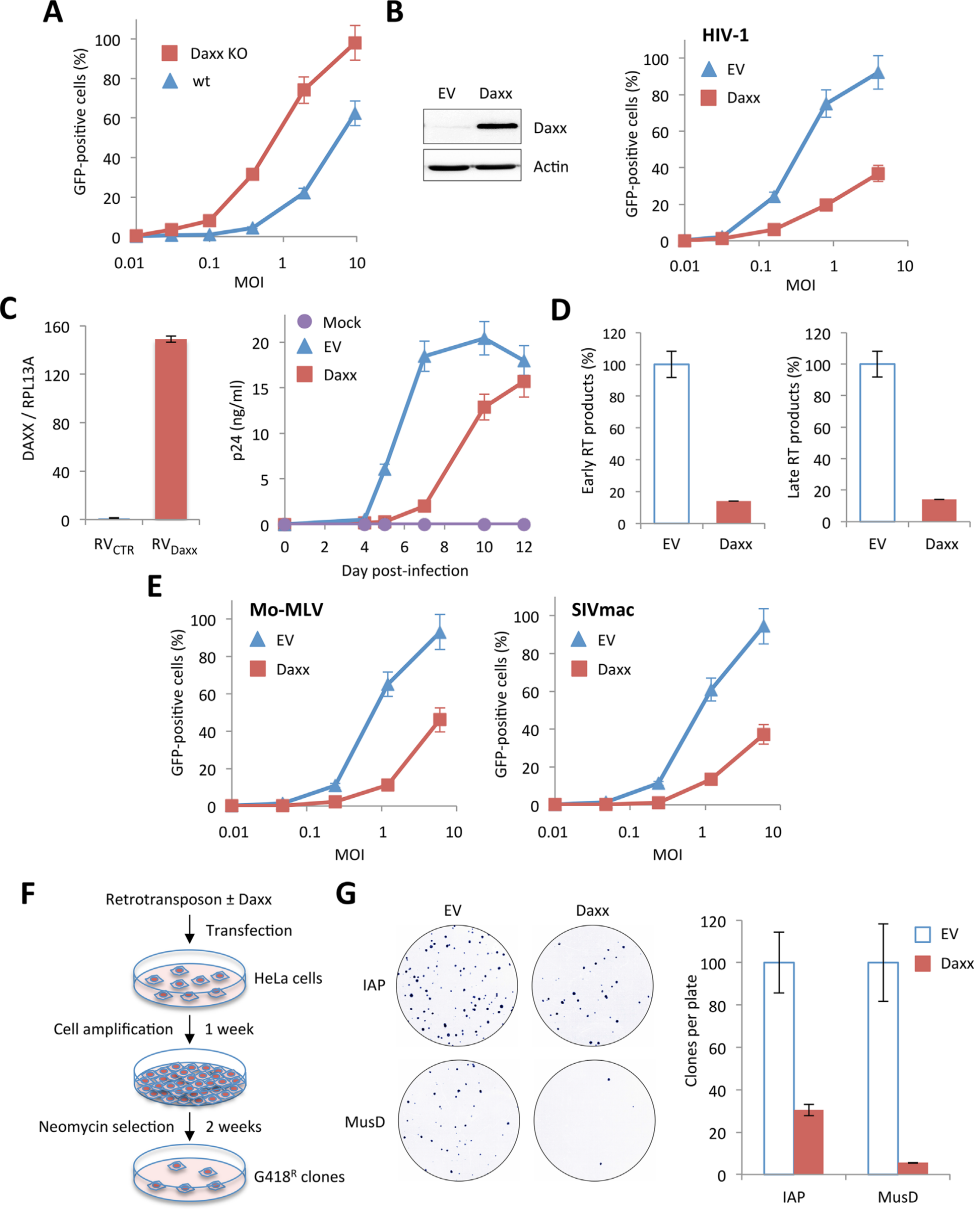
Inhibition of retroviral infections and retro-transposition by Daxx. (A) Wt and Daxx KO MEFs were transduced with increasing doses of HIV-1 vector and the percentage of GFP-expressing cells was determined 48 h later by flow cytometry. Means +/- SD of a typical experiment are shown. Similar results were obtained in three independent experiments. (B) Wt HeLa cells were transfected with an empty (EV) or a Daxx-encoding plasmid. Two days post-transfection, Daxx expression was evaluated by Western-blot analysis (left panel) and the percentage of GFP-expressing cells was determined by flow cytometry 48 h post-transduction with increasing doses of HIV-1 vector (right panel). Graph shows means of triplicate values +/- SD, representative of two independent experiments. (C) PBMCs of healthy adult individuals were activated by anti-CD3/anti-CD28 for 48 h and were subsequently transduced with either a non-coding control (RV_CTR_) or a Daxx-expressing (RV_Daxx_) MLV-derived retroviral vector. Relative expression of Daxx was estimated by RT-qPCR analysis performed two days post-transduction, and expressed as a ratio to RPL13A mRNA expression. Two days post transduction, activated PBMCs were infected with HIV-1 NL4.3 at a dose corresponding to 0.1 ng of p24 and virus replication was followed at given time points post-infection by measuring the amount of HIV-1 p24 by ELISA on the culture supernatants. Results show the mean of triplicates +/- SD and are representative of two independent experiments. (D) Wt HeLa cells were transfected with an EV or a Daxx-encoding plasmid. Two days later, cells were transduced with VSV-pseudotyped HIV-1 vector for 6 h and early and late reverse transcript were quantified by qPCR. (E) Wt HeLa cells transfected with an EV or a Daxx-encoding plasmid were transduced 48 h later with GFP-expressing VSV-G pseudotyped Mo-MLV or SIVmac vectors. The percentage of GFP-expressing cells was determined by flow cytometry. Graphs show means of triplicate values +/- SD, representative of three independent experiments. (F) Experimental procedure for the detection of retrotransposition. (G) Analysis of the activity of Daxx on IAP and MusD retrotransposons. Retrotranspositions (defined by quantifying G418R clones) are presented as percentages relative to samples containing retroelement alone. Results show the mean of three independent experiments +/- SD.

In order to test whether Daxx is involved in HIV-1 restriction, we first tested the capacity of Daxx KO MEFs to be transduced by the HIV-1 vector, compared to wt MEFs. As observed in the case of PML (Fig 2A), the absence of Daxx led to a significant enhancement of the proportion of GFP-positive cells (Fig 6A). However, in contrast to PML whose overexpression had no effect on HIV-1 infection, transfection of a Daxx-encoding plasmid in HeLa cells led to a dramatic diminution of HIV-1 transduction efficiency (Fig 6B), demonstrating that Daxx is able to counteract an early step of HIV-1 replication. We confirmed this observation on primary cells by transducing activated PBMCs with a retroviral vector expressing Daxx, followed by their infection with 0.1 ng p24 of HIV-1 NL4.3. RT-qPCR analysis performed two days post-transduction revealed that Daxx mRNA expression was enhanced by approximately 150-fold (Fig 6C, left panel). The kinetics of HIV-1 replication was followed for 12 days by measuring the p24 protein by ELISA in the supernatant every 2 days. As shown in Fig 6C (right panel), HIV-1 spreading infection was significantly inhibited in Daxx-overexpressing cells, further confirming that Daxx expression interferes with HIV-1 infection. These observations strongly suggest that the inhibition of HIV-1 by PML was in fact due to Daxx. If this is true, then Daxx should be able to block HIV-1 RT and to inhibit other retroviruses, such as MLV or SIVmac.

In order to verify this hypothesis, we first investigated at what step of HIV cycle Daxx interferes. For this, HeLa cells transfected with Daxx or with an empty vector were transduced with HIV-1 and early and late RT products were quantified by quantitative PCR 6 h post-transduction. As shown in Fig 6D, Daxx overexpression promoted a 10-fold decrease in the amount of both early and late RT products, suggesting that Daxx prevents the accumulation of RT products.

Next, HeLa cells overexpressing or not Daxx were transduced with GFP-encoding Mo-MLV or SIVmac. As expected, Daxx expression led to a marked inhibition of both retroviruses (Fig 6E). We did not test EIAV, as it is efficiently blocked by TRIM5α in human cells [3].

Since LTR retrotransposons, also called endogenous retroviruses (ERVs), also rely on a cytoplasmic reverse-transcription step for spreading, we sought to investigate whether Daxx could block their retrotransposition. Most ERVs have lost their capacity to replicate but several, including some murine intracisternal A-particle (IAP) and MusD sequences are still active. These elements can be transcribed into RNA, reverse transcribed into DNA in the cytoplasm and then integrated into the genome at a new location. In order to test the capacity of Daxx to interfere with ERV retrotransposition, we used a cell-based retrotransposition assay, in which the retroelement is marked with a neo cassette, which consists of the neo gene cloned in reverse orientation and rendered inactive by the presence of a forward intron. This intron is spliced out of the RNA intermediate, resulting in a functional gene after RT and integration [34,35]. We tested two LTR retrotransposons: IAP and MusD. HeLa cells were transfected with the marked retroelements, together with or without a Daxx-encoding plasmid. Transfected cells were allowed to grow for 1 week and seeded for G418 selection. After two weeks of selection, foci were fixed, stained and quantified (Fig 6F). Daxx potently inhibited both IAP and MusD retrotransposition, resulting in a 4-fold and 20-fold decrease in the number of G418R clones, respectively (Fig 6G), confirming that Daxx is a broad-spectrum inhibitor of retroviral RT.

### RT inhibition by Daxx requires PML CB formation

**Fig 7:**
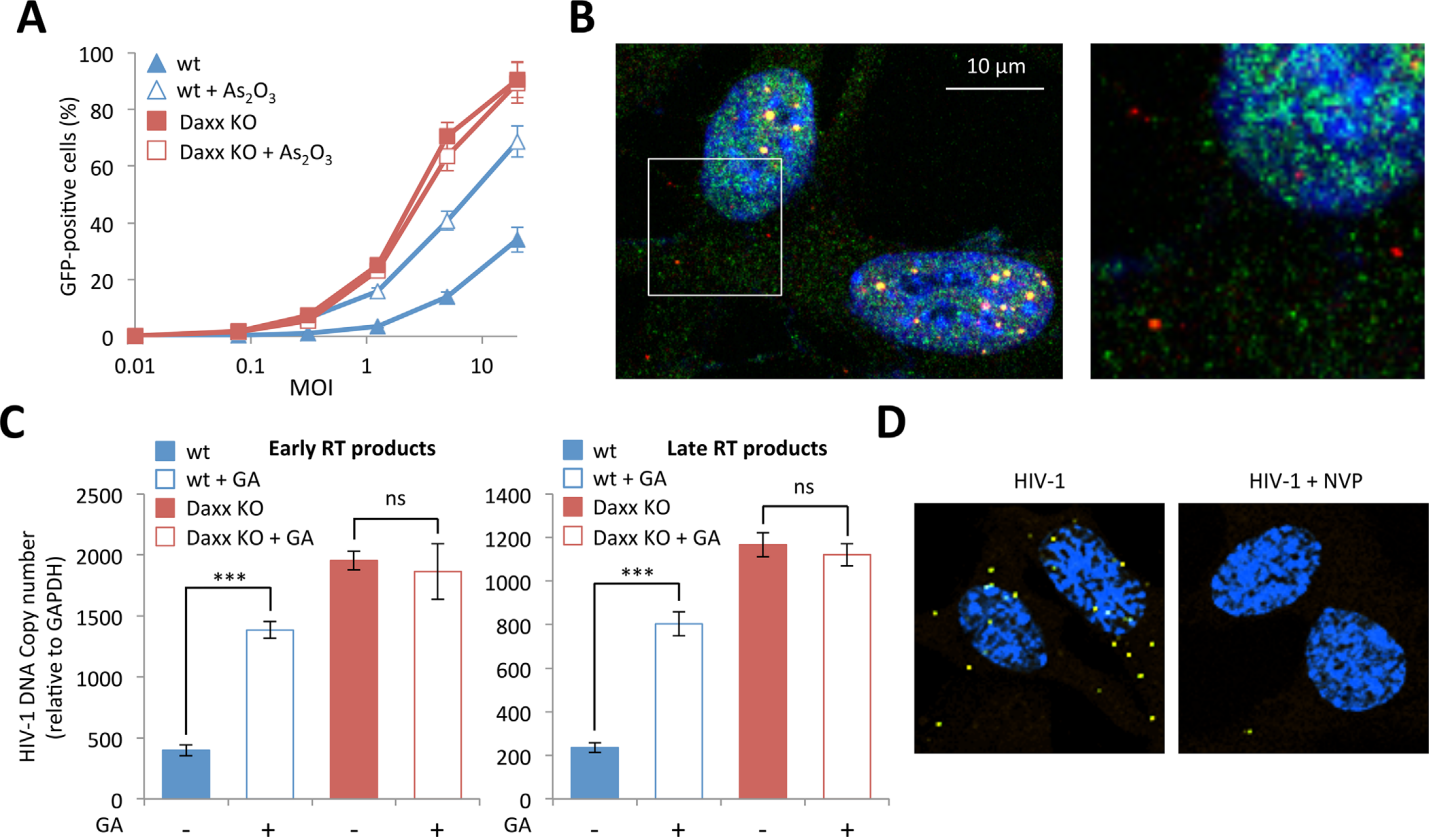
(A) Wt or Daxx KO MEFs were treated with 2.5 μM of As_2_O_3_ during 24h and cells were infected at different MOI with HIV-1 vector during 48h. The percentage of GFP expressing cells was determined by flow cytometry. The graph shows means of duplicate values +/- SD, representative of three independent experiments. (B) Wt HeLa cells were transduced for 2 h with HIV-1 vector and PML (in red) and Daxx (in green) localization was analyzed by immunofluorescence. (C) Wt or Daxx KO MEFs were transduced with HIV-1 vector in the presence or not of 100 μM of GA. Six hours post-transduction, early and late reverse transcripts were quantified by qPCR. Graphs show means of triplicate values +/- SD, representative of two independent experiments. Student t-test was performed to determine P values (***P<0.001; ns, not significant). (D) Wt MEFs were treated or not with nevirapine (NVP), transduced with HIV-1 for 2 h and probed with anti-Daxx and anti-HIV-1 p24 antibodies. Representative images of a Duolink experiment are shown.

Having shown that Daxx is able to restrict retroviral infection by preventing the accumulation of RT products, we sought to determine whether Daxx requires PML in order to display its antiretroviral activity. To do this, we first evaluated the effect of As_2_O_3_ treatment on HIV-1 infection in Daxx KO MEFs. If Daxx's restriction activity is independent of PML, As_2_O_3_ treatment should still enhance HIV-1 infectivity in Daxx KO cells, whereas it should have no effect if Daxx and PML act cooperatively. To answer this question, we treated wt or Daxx KO MEFs with As_2_O_3_ and transduced them with the HIV-GFP vector. As previously shown, arsenic increased the capacity of wt MEFs to be transduced (Fig 2C), but had no effect on HIV transduction of Daxx KO MEFs (Fig 7A), thus confirming that Daxx is the mediator of PML-dependent restriction.

Next, we wanted to evaluate the implication of PML in Daxx's restriction activity and in particular the role of retroviral-induced PML CBs. Since we found that Daxx colocalizes with incoming HIV-1 cores whereas cytoplasmic PML does not, it is highly improbable that the two PML NB partners interact in the cytoplasm upon retroviral infection. Since we cannot immunostain HIV-1 p24, PML and Daxx simultaneously due to antibody incompatibilities, we studied the localization of Daxx and PML in wt MEFs presenting PML dots in the cytoplasm, which was considered as a hallmark of HIV-1 infection. As anticipated, while there is a clear nuclear colocalization of PML and Daxx in PML NBs, cytoplasmic Daxx does not reside within PML CBs (Fig 7B). Although the formation of PML CBs could be unrelated to Daxx mediated retroviral restriction, and despite the fact that PML and Daxx do not colocalize in the cytoplasm of infected cells, we decided to investigate whether retroviral-induced PML CBs are implicated in Daxx mediated retroviral restriction.

Since we showed that the formation of PML CBs requires the SUMOylation machinery (Fig 3C), we evaluated the effect of GA on Daxx-induced HIV-1 restriction. If Daxx's restriction activity depends on the formation of PML CBs, it can be expected that GA treatment would enhance HIV-1 infectivity in wt cells but will have no effect in cells depleted for Daxx. To test this hypothesis, we treated wt and Daxx KO MEFs with GA, transduced them with HIV-1 for 6h and determined the amount of RT products by quantitative PCR. As anticipated, whereas the amount of RT products was significantly enhanced in wt MEFs upon GA treatment, it was unaffected in Daxx KO MEFs (Fig 7C). This observation further confirms that Daxx is the mediator of the PML-dependent inhibition of HIV-1 reverse-transcription.

Finally, we tested whether inhibiting RT would prevent the recruitment of Daxx to incoming retroviral capsids. To do this, we performed Duolink *in situ* PLA on wt MEFs transduced with HIV-1 in the presence or not of NVP. Cells were then probed with anti-Daxx and anti-HIV-1 p24 primary antibodies, followed by species-specific PLA probes. As shown in Fig 7D, NVP treatment inhibited the recruitment of Daxx in the vicinity of incoming p24. Since NVP also prevented HIV-induced PML CB formation (Fig 3B), this result further confirms that the formation of PML CBs triggered by retroviral RT is a prerequisite for RT inhibition by Daxx. Altogether, these observations show that even if PML and Daxx do not colocalize in the cytoplasm of retrovirus-infected cells, restriction of infection by Daxx is dependent on PML redistribution.

### PML is required for Daxx protein expression

**Fig 8:**
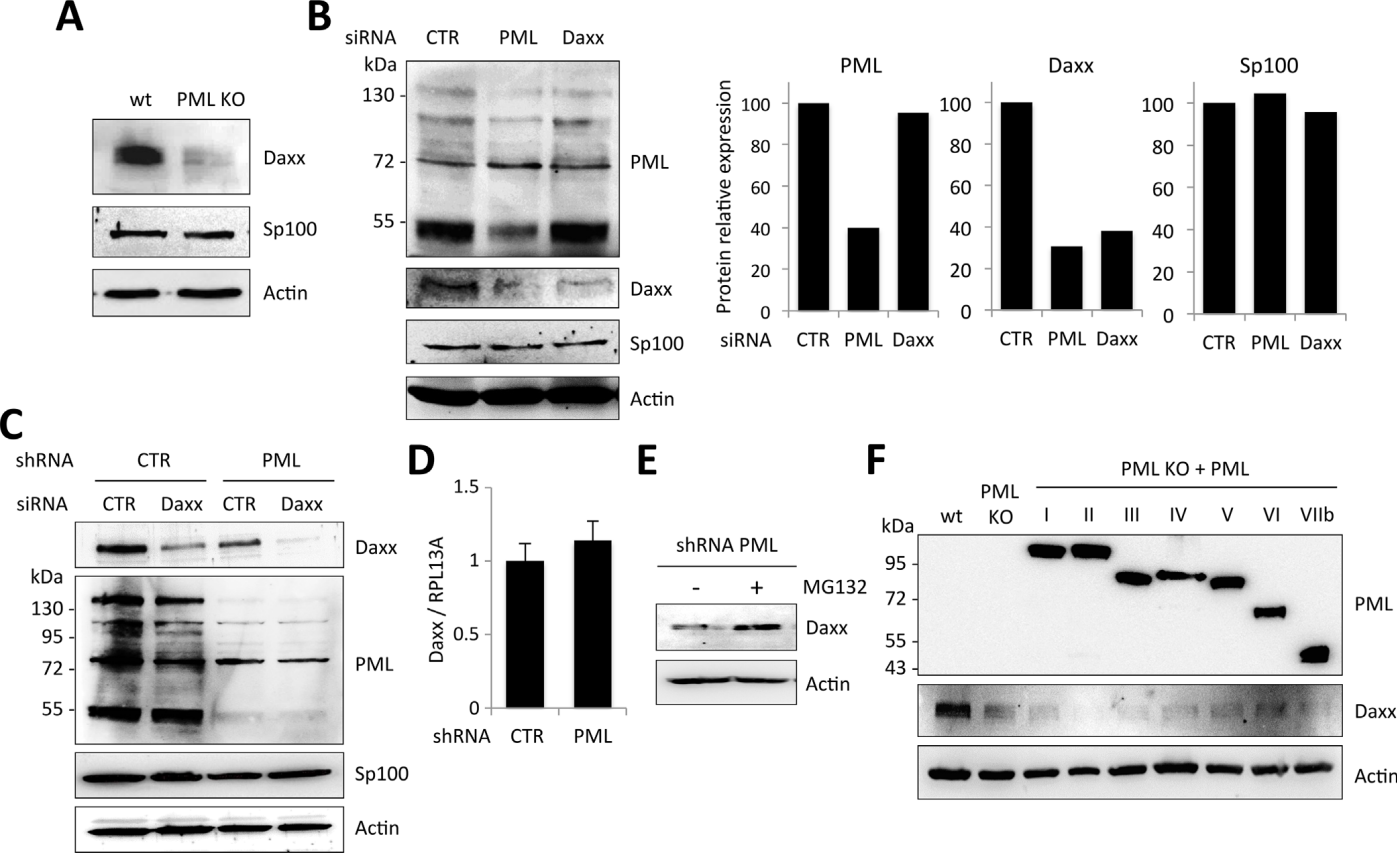
PML expression is required for Daxx stability. (A) Western-blot analysis of Daxx, Sp100 and actin expression in wt and PML KO MEFs. (B) Western-blot analysis of PML, Daxx, Sp100 and actin expression in wt HeLa cells transfected with non-targeting control (CTR), PML-specific or Daxx-specific siRNA. Intensity of individual bands was quantified using ImageJ software. (C) HeLa cells stably expressing a non-targeting control shRNA (CTR) or a PML-targeting shRNA were transfected with CTR or Daxx-specific siRNA. Expression of Daxx, PML, Sp100 and actin were evaluated by western-blot analysis. (D) Daxx mRNA relative expression in HeLa cells expressing CTR or PML-targeting shRNA, expressed as a ratio to RPL13A mRNA expression. (E) HeLa cell expressing PML-targeting shRNA were treated with MG132 for 6 h and analyzed by western-blot for Daxx and actin expression. (F) Western-blot analysis of PML, Daxx and actin expression in wt, PML KO and PML KO MEFs stably expressing each human PML isoform (PMLI to PMLVIIb). Note that endogenous PML was not detected in wt MEFs because the western-blot was revealed with anti-PML antibodies specific to human PML isoforms.

Having shown that Daxx is the mediator of retroviral restriction in PML expressing cells, we next asked why PML knockdown prevents Daxx-mediated inhibition of retroviral infections. We started by looking at Daxx expression in wt and PML KO MEFs. Unexpectedly, Daxx protein expression was dramatically reduced in PML KO compared to wt MEFs, whereas Sp100 protein level was not affected (Fig 8A). To confirm this observation in human cells, we depleted either PML or Daxx expression using siRNA in HeLa cells. Surprisingly, the siRNA directed against PML reduced the expression of PML, as anticipated, but also drastically diminished Daxx expression, as efficiently as the Daxx-targeting siRNA (Fig 8B). We performed another experiment where we silenced Daxx in HeLa cells stably expressing a non-targeting control shRNA or a shRNA targeting PML. Once again, shRNA-mediated PML knockdown dramatically reduced the expression of Daxx. Furthermore, whereas Daxx can still be detected in cells transfected with a siRNA targeting Daxx, it is completely depleted in cells expressing the shRNA targeting PML (Fig 8C).

Altogether, these observations strongly suggest that Daxx expression requires PML. In order to test whether this effect of PML on Daxx expression is transcriptional, we quantified Daxx mRNA by RT-qPCR in HeLa cells stably expressing the control or the PML-specific shRNA. Our results indicate that Daxx mRNA expression is not affected by PML knockdown (Fig 8D), suggesting that PML stabilizes Daxx at the protein level. To confirm this, HeLa cells stably expressing PML-specific shRNA were treated or not with the proteasome inhibitor MG132 and Daxx expression was analyzed by western-blot. Whereas Daxx was fairly undetectable in extracts from PML knockdown untreated cells, its expression was restored in extracts from MG132-treated cells, suggesting that absence of PML leads to proteasomal degradation of Daxx (Fig 8E).

From these results, it appears that PML expression prevents Daxx degradation, thus explaining why PML silencing enhances retroviral infection. However, one could have anticipated that the transfection of PML isoforms (Fig 5A) should have restored Daxx expression and thus Daxx-mediated HIV-1 inhibition, which was not the case. In an attempt to understand this apparent discrepancy, we examined PML and Daxx expression in PML KO MEFs overexpressing each human PML isoform. As shown in Fig 8F, none of these PML isoforms was able to restore Daxx expression at a level comparable to wt MEFs. This explains why overexpression of each human PML isoform (PMLI to PMLVIIb) was unable to block HIV-1 transduction and suggests that Daxx protein expression requires the cooperation of different PML isoforms.

## Discussion

In 2001, Turelli *et al*. reported that HIV-1 infection triggers a rapid cytoplasmic translocation of PML [21]. They also reported that As_2_O_3_ treatment enhances HIV-1 transduction efficiency, thus suggesting that PML is able to block an early step of viral replication cycle [21]. However, another report later showed that wt and PML KO MEFs were equally transduced with an HIV-derived vector and that As_2_O_3_ could enhance transduction efficiency in both cell types, thus concluding that the effect of arsenic on HIV-1 transduction is independent of PML degradation [22].

In this report, we re-examined the role of PML in HIV-1 replication. Our results are essentially in agreement with Turelli *et al*., as in our hands, HIV-1 transduction was much more efficient in PML KO MEFs than in wt MEFs (Fig 2A). Furthermore, whereas As_2_O_3_ treatment restored HIV-1 infectivity in wt MEFs, it had no effect in PML KO cells (Fig 2, panels B and C). Furthermore, we also observed the rapid formation of PML bodies in the cytoplasm upon HIV-1 infection.

From these observations that were previously published, we investigated further what triggers the formation of PML bodies in the cytoplasm of infected cells and investigated whether PML itself is a restriction factor. We first showed that RT of viral RNA is a prerequisite for PML translocation, since treatment with nevirapine prevented the formation of PML CBs. In addition, using an inhibitor of SUMOylation, we showed that as for PML NBs, HIV-induced PML CB formation is dependent on SUMOylation. Interestingly, not only HIV-1 was found to trigger the formation of PML CBs but also other retroviruses such as SIVmac, MLV Moloney and EIAV. Infection with other viruses from distinct viral families such as Sendai virus, Influenza A virus, VSV or EMCV did not lead to PML re-localization. This result demonstrates that the formation of PML CBs is not the consequence of a cellular stress associated with infection, but a retrovirus-specific event. Altogether, these results strongly suggest that PML CB formation is triggered by the synthesis of RT products at a very early step of retroviral infection. It has to be noted that PML cytoplasmic translocation has previously been observed with other viruses, such as lymphocytic choriomeningitis virus (LCMV) and rabies virus [36,37]. However, in these two cases, PML re-localization is a late event of viral replication, whereas it can be observed as early as 30 min in the case of retroviral infections. Independently of viral infections, the presence of PML in the cytoplasm has previously been reported. Indeed, although PML is predominantly nuclear, cytoplasmic functions of PML have recently emerged [38,39]. For instance, the cytoplasmic PML isoform cPML3Δ3–7 is required for the interaction of Smad2/3 with SARA and is therefore an essential modulator of TGF-beta signaling [40]. Another example is the generation of a cytoplasmic alternatively spliced PML isoform during herpes simplex virus type 1 (HSV-1) infection [41]. Interestingly, this particular PML isoform termed PMLIb or cPMLΔ5–6 confers resistance to HSV-1 by sequestering the immediate early HSV-1 protein ICP0 in the cytoplasm [41], demonstrating for the first time a cytoplasmic PML isoform displays an antiviral activity.

In the case of retroviruses, we show in this report that PML knockdown enhances infection by HIV-1, EIAV, Mo-MLV and SIVmac. Furthermore, we show that HIV-1 RT efficiency is highly increased in PML KO cells, which further suggested that PML could interfere with the RT of retroviruses. However, despite these observations, overexpression of each human PML isoform in PML KO MEFs did not have any effect on retroviral transduction, suggesting that even if PML is involved in this phenotype, it is not the direct mediator of retroviral restriction.

We demonstrate that rather than PML, Daxx is the cellular protein that confers resistance to retroviral infections. First, unlike PML and Sp100, Daxx colocalizes with HIV-1 p24 in the cytoplasm of infected cells. Furthermore, we show that whereas Daxx knockdown significantly enhances HIV-1 transduction, Daxx overexpression leads to a dramatic inhibition of HIV-1 transduction in HeLa cells and also inhibits HIV-1 propagation in PBMCs. We demonstrate that Daxx interferes with an early step of HIV-1 replication by preventing the accumulation of reverse-transcripts. Interestingly, inhibition of HIV-1 RT by NVP not only prevents the formation of PML CBs upon HIV-1 infection but also prevents the interaction of Daxx with incoming HIV-1 p24, strongly suggesting that the two events are linked. This was further demonstrated by the fact that As_2_O_3_ treatment, which promotes PML degradation and enhances HIV-1 infection in wt cells, is unable to increase HIV-1 transduction efficiency in Daxx KO MEF cells. Furthermore, we showed that GA treatment, which prevents the formation of PML CBs, enhances the efficacy of RT in wt cells but has no effect in Daxx KO MEFs. Altogether, these observations support the fact that PML and Daxx act cooperatively to interfere with HIV-1 infection and that the formation of PML CBs is necessary for Daxx to inhibit the formation of RT products.

Finally, we found that Daxx restriction activity is not limited to HIV-1, as it can interfere with other retroviruses, such as MLV Moloney or SIVmac. Importantly, Daxx overexpression also inhibits retrotransposition of murine ERVs, demonstrating for the first time that this cellular protein is a broad inhibitor of RT.

Daxx is one of the few cellular proteins with PML, Sp100 and ATRX that are constitutive residents of PML NBs. Daxx localization within PML NBs is mediated through its direct recruitment by PML [24,42]. PML SUMOylation was shown to be necessary for this interaction [24,42], presumably because SUMOylated PML is recognized by the SUMO-interacting motif (SIM) identified in the carboxy-terminus of Daxx [43].

However, although PML NBs constitute preferential depositories of Daxx, the protein has alternative cellular localization both in the nucleus and in the cytoplasm [44]. Daxx was initially identified by a yeast two-hybrid screen as a binding partner for the death domain of the Fas receptor [45]. In PML KO cells, Daxx was shown to be mostly associated with condensed heterochromatin [24]. Remarkably, we show here that the expression of Daxx is dramatically decreased in the absence of PML. This was observed in PML KO MEFs, which express very little amount of Daxx compared to wt MEFs and also in HeLa cells where PML expression was reduced by RNA silencing. Given the fact that Daxx mRNA expression was not affected in PML-depleted cells and that MG132 prevented Daxx downregulation, it is likely that PML expression prevents proteasome-dependent Daxx degradation. This would reinforce the notion that PML NBs serve as nuclear depots for Daxx, where PML serves as a protective shell for Daxx and other PML NB resident proteins [25,44]. It has to be noted, however, that in infected cells, Daxx was not found to reside in or next to PML CBs, suggesting that only nuclear PML might be able to protect Daxx from degradation.

In addition to PML, Daxx has been shown to interact with a plethora of other proteins implicated in various cellular processes. Beyond its implication in cell death, which is still under debate, the most obvious function of Daxx is transcriptional repression, which is achieved through the interaction of Daxx with several nuclear proteins implicated in histone deacetylation, chromatin remodeling, DNA methylation or transcription [44].

Daxx has also been implicated in antiviral defense and as a consequence, many viral proteins target Daxx to favor gene expression and replication [46]. Among retroviruses, only one case of Daxx-mediated inhibition has been reported. It is the case of avian sarcoma virus since Daxx was found to interact with the viral integrase and to inhibit viral transcription by inducing DNA methylation and histone modifications on viral chromatin [47,48]. In this paper, we report a new inhibition mechanism used by Daxx to interfere with retroviral replication. This is, to our knowledge the first report showing that Daxx inhibits reverse transcription. Among the many questions raised by our study, it would be of great interest to determine whether Daxx physically interacts with reverse-transcription complexes and, if this is the case, which protein is targeted. Given the fact that most of Daxx's targets are SUMOylated, possible candidates in the case of HIV-1 could be p6 [49] or integrase [50], which have both been shown to constitute SUMO substrates. Interestingly, both HIV-1 p6 and integrase have been previously shown to interact with Daxx [49,51]. However, since Daxx also inhibits other retroviruses such as MLV, SIVmac and EIAV and also interferes with retrotransposition, it may rather target a cellular protein involved in RT, such as HDAC1, Gemin2 or AKAP149 [52]. One other question that remains to be addressed is what event triggers PML translocation upon retroviral infection. We showed that the appearance of PML in the cytoplasm is a rapid event and that it requires reverse-transcription. The fact that PML NB main constituents, PML, Sp100 and Daxx, are all found in the cytoplasm of infected cells may suggest that PML, as the organizer of PML NBs, is the mediator of this global re-localization. Since PML re-localization begins as early as 30 min post-infection, this event is therefore most likely signal mediated, as suggested by Turelli et al. [21]. In the case of HIV-1, two sensors of RT products have been identified so far, cGAS and IFI16 [53,54]. However, recent studies have demonstrated that HIV-1 has developed the capacity to escape innate sensing in myeloid cells through the cloaking of reverse-transcripts by viral capsid and cofactor proteins [55–57]. Our observations reveal that in some cells at least, RT products can be detected by the cell at a very early stage, and therefore suggest that reverse-transcripts may be not as efficiently protected by the capsid shield. It would be of interest to study PML localization in dendritic cells during HIV-1 infection in order to determine whether RT triggers the formation of PML CBs or whether RT products are particularly efficiently shielded in these cells.

Further investigations will be required to understand how retroviral reverse-transcription triggers the re-localization of PML and PML NB resident proteins, leading to Daxx-mediated retroviral restriction.

## Material and Methods

### Reagents and antibodies

Ginkgolic acid (GA) was purchased from Merck Millipore and was used at 100 μM. Nevirapine (NVP) was purchased from Sigma Aldrich. Transfections were performed using FuGene 6 transfection reagent (Promega). Human PML was detected using rabbit anti-PML H-238 (Santa Cruz sc-5621), whereas murine PML was detected using mouse anti-PML 36.1–104 (Millipore). Rabbit anti-Daxx M-112 antibodies were purchased from Santa-Cruz (sc-7152). Rabbit anti-Sp100 antibodies were a gift from Hans Will (Hamburg, Germany). Mouse monoclonal anti-p24 clone 183-H12-5C and rabbit anti-p24 (HIV-1-SF2 p24 Antiserum) were provided through the NIH AIDS Reagent Program. Actin was detected using HRP-conjugated monoclonal anti-Actin antibody (Sigma). For immunofluorescence analyses, secondary antibodies conjugated to Alexa Fluor were purchased from Molecular Probes. For western-blot analyses, HRP-conjugated secondary antibodies were goat anti-mouse IgG (Santa-Cruz, sc-2055) and goat anti-rabbit IgG (Cell Signaling #7074).

### Cells, viruses and retro-/lentiviral vectors

HeLa cells, HEK 293T cells, murine embryonic fibroblasts (MEFs) derived from wt, PML KO mice (kindly provided by Pier Paolo Pandolfi)[29] or Daxx KO mice (kindly provided by Gerd Maul)[58] and Mus Dunni Tail Fibroblasts (MDTF) were cultured in Dulbecco’s Modified Eagle Medium (Life Technologies) supplemented with 10% fetal calf serum, 2 mM L-glutamine, 1 mM sodium pyruvate, 10 U/mL Penicillin and 10 μg/mL streptomycin. HIV-1 NL4.3 virus was produced by transient transfection of HEK 293T cells using calcium phosphate precipitation. Viruses were harvested at 48 h after transfection and viral yield was measured by p24 ELISA according to the manufacturer's instructions (PerkinElmer Life Sciences). Vesicular stomatitis virus glycoprotein (VSV-G)-pseudotyped retroviral vectors were generated by co-transfecting HEK 293T cells with pVSV-G, a Gag-Pol expression plasmid and green fluorescent protein (GFP)-expressing retroviral vector, using the ProFection calcium phosphate kit (Promega). Mo-MLV vectors were produced using pCFG2-eYFP and pHIT60. HIV-1 vectors were prepared with pCSGW and p8.91. SIVmac vectors were generated with pAd-SIV3+ and GAE-CMV-GFP, which were kindly provided by F.L. Cosset (ENS Lyon, France). EIAV vectors were produced using the ONY8.9 construct (Oxford Biomedica). All viral stocks were titrated on MDTF cells and analyzed by flow cytometry. The multiplicity of infection (MOI) is defined as MOI = -2 ln(1-fp), where fp is the fraction of GFP/YFP-positive MDTF cells. Cells were transduced with increasing doses of retroviral vectors and the percentage of GFP-expressing cells was determined by flow cytometry 48 h post-transduction and analyzed using FlowJo software (Treestar). The lentiviral vector expressing a PML-specific shRNA (pLV-PMLi) and its non-targeting control pLV-shCon were kindly provided by Yasuo Ariumi [59]. HeLa cells were transduced with the vectors and selected for 2 weeks under puromycin selection (1 μg/ml). The retroviral construct pLNCX2-Myc-hDaxx was a gift from Armelle Corpet [60]. Lentiviral vectors encoding human PML isoforms (PMLI to PMLVIIb) were constructed by inserting each ORF in the HIV-1-derived pTRIP vector, provided by Pierre Charneau (Institut Pasteur, Paris). For retrotransposition experiments, plasmids encoding IAP (pGL3-IAP92L23neoTNF) and MusD (pCMVMus-6DneoTNF) retrotransposons were kindly provided by Thierry Heidmann (Institut Gustave Roussy, Villejuif, France)[34,35].

### Immunofluorescence analyses

Cells were grown on 12mm slides and fixed with 4% paraformaldehyde for 15 min, rinsed in PBS, incubated in NH_4_Cl 50 mM for 10 min and permeabilized with 0.5% BSA / 0.3% Triton X100 / 2% Normal Goat Serum for 30 min. Cells were then incubated with primary antibodies at room temperature for 30 min. Slides were rinsed in PBS 0.5% BSA and incubated at RT in the dark for 30 min with secondary antibodies and rinsed in PBS 0.5% BSA. Finally, slides were washed in PBS, counterstained with Hoechst 33342 and mounted in Fluoromount-G medium. Images were digitally acquired with a Zeiss LSM 710 confocal Microscope. For quantitative analyses of PML CBs, images were converted to binary mode, nuclei were selected and PML CBs were quantified and measured using the “Analyze particles” module of ImageJ software. Student T test statistical analyses were performed using Statistica software. For Duolink Proximity Ligation Assay (PLA), PFA-fixed MEF cells were pre-incubated with blocking agent for 1 h. After washing in PBS, cells were incubated with primary anti-Daxx and anti-HIV-1-p24 antibodies for 1 h in a pre-heated humidity chamber at 37°C. Slides were washed three times in PBS for 10 min and then they were incubated with Duolink PLA probes (Olink Biosciences) diluted in the blocking agent (1:5) for 1 h in a pre-heated humidity chamber at 37°C. Ligation of the connector oligonucleotides, rolling-circle amplification and detection of the amplified DNA products were done with Duolink Detection Reagents Red according to the manufacturer’s instructions. Nuclei were labeled with Hoechst.

### Quantitative PCR and RT-PCR

For real-time PCR experiments, 12 well plates were seeded with 1.5×10^5^ cells per well. The next day, equal amounts of VSV-G pseudotyped retroviral vectors were treated with 250 U/ml Benzonase nuclease (Sigma-Aldrich) for 20 min at 37°C before adding to cell monolayers. Cells were collected at various timepoints after addition of the virus. Total DNA was extracted using DNeasy Blood & Tissue Kit (Qiagen). Real-time PCR reactions were performed in duplicates using Takyon ROX SYBR MasterMix blue dTTP (Eurogentec) on a 7900HT Fast Real-Time PCR System (Applied Biosystems). The following program was used: 3 min at 95°C followed by 35 cycles of 15 s at 95°C, 20 s at 60°C and 20 s at 72°C. Values for each transcript were normalized to expression levels of murine (mGAPDH) or human (hGAPDH) GAPDH transcripts. qPCR analysis of HIV-1 early RT products was carried out using M667/AA55M primers specific for minus-strand strong stop DNA, whereas late RT products were detected using M667/M661 primers, which amplify the RU5-primer binding site 5' noncoding region.

Primer sequences are as followed: mGAPDH-F: 5'-ATGGTGAAGGTCGGTGTGAAC-3', mGAPDH-R: 5'-GAATTTGCCGTGAGTGGAGTC-3', hGAPDH-F: 5'-ACTTCAACAGCGACACCCACT-3', hGAPDH-R: 5'-GTGGTCCAGGGGTCTTACTCC-3', M667: 5'-GGCTAACTAGGGAACCCACTG-3', AA55M: 5'-GCTAGAGATTTTCCACACTGACTAA-3', M661: 5'-CCTGCGTCGAGAGAGCTCCTCTGG-3'.

For RT-qPCR experiments, total RNAs were extracted using RNeasy Mini Kit (Qiagen). RNA samples were converted to cDNA using PrimeScript RT Reagent Kit (Takara Clontech). Values for each transcript were normalized to expression levels of RPL13A (60S ribosomal protein L13a). Primers used for quantification of transcripts by real time quantitative PCR are as followed: PML-F: 5'- ACACCAGTGGTTCCTCAAGCA-3', PML-R: 5'-CTCGGCAGTAGATGCTGGTCA-3', Daxx-F: 5'-TGCTGGATGATGATGACGAAGAT-3’, Daxx-R: 5'- CTCAGAGGAGCTAGGGGCTTC-3’, RPL13A-F: 5'-CCTGGAGGAGAAGAGGAAAGAGA-3', RPL13A-R: 5'-TTGAGGACCTCTGTGTATTTGTCAA-3'.
